# Comment on *On the propagation of errors* by Jaskolski (2013)

**DOI:** 10.1107/S090744491302917X

**Published:** 2013-11-19

**Authors:** Helen Berman, Gerard J. Kleywegt, Haruki Nakamura, John L. Markley

**Affiliations:** a Department of Chemistry & Chemical Biology, Center for Integrative Proteomics Research, Rutgers, The State University of New Jersey, 174 Frelinghuysen Road, Piscataway, NJ 08854, USA; b PDBe, European Molecular Biology Laboratory–European Bioinformatics Institute, Cambridge CB10 1SD, UK; c PDBj, Institute for Protein Research, Osaka University, 3-2 Yamadaoka, Suita, Osaka, 565-0871, Japan; d BioMagResBank, Department of Biochemistry, University of Wisconsin-Madison, Madison, WI 53706, USA

**Keywords:** wwPDB, errors

## Abstract

The wwPDB responds to the article by Jaskolski [(2013), *Acta Cryst.* D**69**, 1865–1866].

## Abstract 2

A response to the article by Jaskolski [(2013), *Acta Cryst.* D**69**, 1865–1866].

In his paper *On the propagation of errors* (2013 ▶), Jaskolski makes several points about the annotation of ligands in the Protein Data Bank. Since 2007, the Worldwide Protein Data Bank (wwPDB) has undertaken several rounds of remediation of the entire PDB archive in an effort to improve the quality, integrity and consistency of the data. This is an ongoing effort and we will take the issues raised in this paper into account when we prioritize future remediation efforts.

Our response to the specific issues raised is as follows.

*Atom naming.* We agree that the historic practice of using the atom naming of the first deposited ligand of its kind is not serving the users as well as it should. Since PDBx/mmCIF will become the official distribution format for the PDB, it will be possible to have both the officially sanctioned naming convention and any naming scheme used by the depositor. We will discuss the issue of atom naming with CCDC with whom we already collaborate on ligand validation.
*Protonation states*. These depend on the pH of crystallization as well as the microenvironment of the chemical compound or moiety. If the depositor is confident about the protonation state of one or more compounds, residues or moieties, it would in principle be possible to collect this information during deposition.
*Restraint dictionaries*. The wwPDB has set up a PDBx/mmCIF Working Group for the implementation of PDBx/mmCIF in current structure-determination software packages. This group is currently addressing the issue of standardization of restraint dictionaries.
